# A Review of Complicated Cataract in Retinitis Pigmentosa: Pathogenesis and Cataract Surgery

**DOI:** 10.1155/2020/6699103

**Published:** 2020-12-21

**Authors:** Yingying Hong, Hongzhe Li, Yang Sun, Yinghong Ji

**Affiliations:** ^1^Eye Institute and Department of Ophthalmology, Eye & ENT Hospital, Fudan University, Shanghai 200031, China; ^2^NHC Key Laboratory of Myopia (Fudan University), Key Laboratory of Myopia, Chinese Academy of Medical Sciences, Shanghai 200031, China; ^3^Shanghai Key Laboratory of Visual Impairment and Restoration, Shanghai 200031, China; ^4^The First Affiliated Hospital of Dali University, Dali 671000, China

## Abstract

Retinitis pigmentosa (RP) is a set of inherited retinal degenerative diseases that affect photoreceptor and retinal pigment epithelial cells (RPEs), possibly associated with some ocular complications, including cataract. The complicated cataract formation is most likely the result of RP-related inflammation response, and the most common morphology category is posterior subcapsular cataract (PSC). Despite the absence of curative pharmacologic treatment, phacoemulsification with intraocular lens implantation to deal with opacification in the lens is preferred due to the considerable visual outcomes. However, the incidence of intraocular and postoperative complications is higher in RP patients than those without, including intraoperative phototoxic retinal damage, posterior capsular opacification (PCO), capsular contraction syndrome (CCS), pseudophakic cystoid macular edema (PCME), increased postoperative intraocular pressure (IOP), and intraocular lens (IOL) dislocation. Hence, it needs much attention to surgery progress and close follow-up. In this review, we discuss the current understanding of RP patients with complicated cataracts from morphology to potential pathogenesis to cataract surgical procedure and provide a concise description and the recommended management of related surgery complications to broaden the knowledge and lower the latent risks to yield better clinical outcomes.

## 1. Introduction

**Figure 1:**
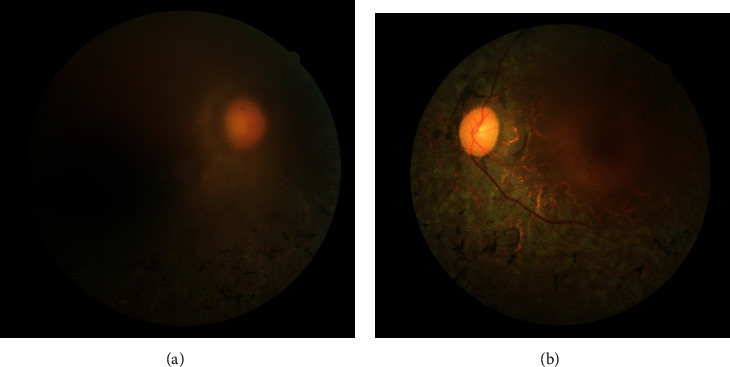
Example of a patient with unilateral retinitis pigmentosa. (a) The right eye showed blurred fundus due to cataract. (b) The left eye showed a typical fundus picture of a RP patient, including bone spicules, attenuated vessels, and waxy pallor of the optic nerve.

The most common inherited retinal dystrophy is retinitis pigmentosa (RP), affecting more than 1.5 million patients with a prevalence of approximately 1 : 4000 worldwide, and primary symptoms are reduced night vision and chronically progressive vision field loss due to photoreceptor cell death [1]. RP can be a typical and syndromic form accompanied by other ocular disorders [1]. The fundus is usually characterized by bone spicules, attenuated vessels, and waxy pallor of the optic nerve as important signs of typical RP (Figure 1) [1, 2].

According to the inheritance patterns, the RP can sort to autosomal dominant RP (ADRP), autosomal recessive RP (ARRP), x-linked RP (XLRP), digenic RP, and mitochondrial RP [2]. More than 80 genes have been found associated with nonsyndromic RP [1] since 1990 when Dryja et al. [3] reported the first identified gene of rhodopsin (RHO) in ADRP. Although the understanding of genetic patterns and pathological mechanism is continuously advancing, there are still no adequate, effective treatments to reverse the disease's visual loss [1, 2, 4]. The possible explanation of reduced vision in RP can be progressive photoreceptor cell death and treatable complications of RP, including cataract, cystoid macular edema (CME), and epiretinal membrane (ERM) [5]. Liew et al. [5] reported that RP complications had a high possibility of bilateral development (>70%).

Cataracts are recognized as the most common anterior segment complication of RP [2]. The opacity of lens generally happens at the mid stage of the disease course with obvious clinical symptoms and signs [4], and the major symptom is glare, especially in bright light [6]. Based on the constrict visual field, loss of transparency in the eyes with RP only in a small part of the lens can lead to disproportionate visual acuity reduction [7]. Furthermore, compared with the mean age at surgery of 72–74 years for age-related cataract (ARC) [8], the surgery time in RP is earlier, with an average age at surgery of 47–63 years old in previous studies [6, 8–13] and 83% of the patients with mean age at surgery between 30 and 59 years in the study of Bastek et al. [14]. The decreased visual acuity with continuous development of cataracts severely influences the patient's life quality. In addition, there are still no effective and curative pharmacologic treatments for patients with cataracts [15]. Despite the potentially poor results of re-impaired visual acuity caused by the surgery complications, including phototoxic retinal damage during the surgery, postoperative posterior capsular opacification (PCO), capsular contraction syndrome (CCS), pseudophakic cystoid macular edema (PCME), increased intraocular pressure (IOP), and intraocular lens (IOL) dislocation [6, 8, 10, 12, 13, 16, 17], significant improvement of visual acuity and subjective symptoms in the majority of patients were also published in many studies [6, 8–14, 16, 18, 19].

## 2. Complicated Cataract

The morphology of cataract in RP patients includes posterior subcapsular cataract (PSC), anterior polar cataract (APC), and nuclear cataract (NC) [20–22]. Although the data are limited, the prevalence of cataract among the RP patients from different areas is substantially different according to the studies from the United States [23], Italia [24], and England [5]. In addition, Liew et al. [5] found that patients with cataract were also less likely to have CME, suggesting different pathophysiological processes among them, especially the status of the retinal tissue.

### 2.1. Posterior Subcapsular Cataract

**Figure 2:**
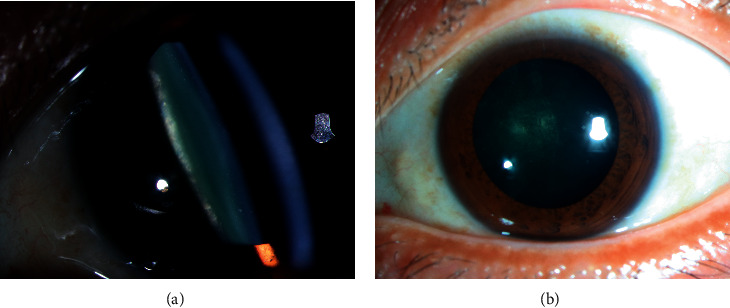
Example of a patient with posterior subcapsular cataract (PSC) in retinitis pigmentosa. (a) Opacification in slit-lamp image of lateral posterior capsular. (b) Holistic picture of PSC appearing as minor lens opacity in the central part of the posterior pole.

The most common morphology of cataract in RP patients is PSC (approximately 41%–53% frequency [25]). Pruett [23] reported that the rate of cataracts in typical RP was 46.4%, and 93.6% were PSCs. PSC appears as minor lens opacity in the central part of the posterior pole (Figure 2), responsible for losing central vision. Previous studies have reported that the age and genetic type of RP contributed to different rates of PSC, although the conclusions varied from that of different research studies [18, 26, 27]. In addition, the severity of lens opacities might increase with age [18] and disease duration [27]. The data from the study of Fishman et al. [18] showed 41% (patients with ADRP) to 90% (patients with XLRP) probability of PSC formation by age 40 and RP initiating earlier than the general population with ARC estimated to occur in 2.1% of individuals at age 52 to 85 years [12]. However, the PSC was found in similar frequencies among all genetic types of RP in another study with unknown origin [20]. These studies' data showed that the patients with RP were more susceptible and earlier to develop PSC, which might account for initial cataract surgery to maintain the residual central visual acuity as long as possible.

It is known that the intraocular microenvironment may be changed by the inflammatory reaction of RP [28–37] (Table 1), which might be a secondary role in the disease pathogenesis and pose a risk of cataractogenesis and surgery complication, aligned with previous studies that proinflammatory status was also found in uveitis-related cataract [38, 39], high myopic cataract [40], and congenital cataract [41, 42]. For example, Yoshida et al. [31] found increased proinflammatory cytokines and chemokines both in the aqueous humor and vitreous fluid of RP patients, especially monocyte chemoattractant protein-1, which can recruit inflammatory cells functioning as chemoattractants [43] involved in numerous pathological processes [40]. In addition, a more robust inflammatory response in young RP patients was also found in their study, possibly related to early PSC onset in patients [31]. Some experimental [43–45] and clinical studies [33] supported the notion that these increased intraocular cytokines may be produced from the degenerative retinal tissue through the damaging blood-ocular barrier (blood-aqueous and blood-retinal barrier) [46–48].

Many studies showed a close association between PSC formation and inflammatory reaction [25, 34, 49]. Gwon et al. [49] first demonstrated the PSC formation after concanavalin A (a nonspecific inflammatory agent)-induced inflammation in New Zealand Albino rabbits by intravitreal injection. Also, the elevated aqueous flare in RP patients was considered a significant risk factor for PSC formation [25]. However, the cytokines' specific action in cataracts is complicated, and related research had been an underexplored domain. In the future, it is necessary to elucidate the interaction mechanism of these cytokines in cataract deeply.

It is presumed that another mechanism of PSC is vested on oxidative stress. The hypothesis is based on the highly membranous rod outer segments with a high proportion of polyunsaturated lipids susceptible to peroxidation [50]. Zigler et al. studies from ex vivo [50] and in vivo of Royal College of Surgeons (RCS, an animal model of RP) rats [51] showed that the lens damage was correlated with the products of lipid peroxidation. Similarly, a study of RCS rats demonstrated that the activity of glutathione reductase (a substance against oxidation) was reduced in the cataractous lenses [52]. In addition, experimental evidence suggested that cataract formation was associated with the light entering the eyes [53, 54], nutrition [55], and macrophages accumulating in the vicinity of the lens [56], supporting this hypothesis. Moreover, a possible interactive network between oxidative stress and inflammation exists since a laboratory study showed that antioxidant treatment could effectively inhibit the production of proinflammatory cytokines [32], which confirmed the complicated mechanism of PSC in RP.

The following is about the studied pathological process of cataract in RP patients probably associated with these substances. The process of PSC formation is characterized by proliferation of dysplastic bladder-like fibers, or Wedl cells, in the meridional region of the lens that subsequently migrates and aggregates the posterior pole [57]. Abnormal posterior end growth [57] and migration, followed by loss or disruption of normal cytoarchitecture that culminate in lenticular transparency loss, were seen in the RCS rats with PSCs [58, 59]. The observed alteration of the basal membrane complex related to adhesion mechanics might be initiated by inflammation, which can explain this phenomenon [58, 59]. In addition, Kuszak et al. [60] inferred that lens has some self-repair capability of PSC when internalized PSCs were observed in those RCS rats with re-establishment of semblance of the normal lens structure.

Clinically, the vacuolar opacities were observed in PSC of RP patients [61, 62]. A prospective observational study described vacuolar PSC with amoeboid shape in the less opacified area but central in the axis and also observed degenerative focal lens epithelial cells (LECs) in the lesion, which seems to be a forerunner of dysfunction in aberrantly migrated LECs [62]. Consistent with previous studies that found that there were ion pump damage and various alterations of metabolic parameters in PSC patients without RP [63, 64], LEC physiological function disrupted in the lens with RP might also lead to overall impairment of water balance resulting in PSC formation [7, 65]. Another possible source of this liquefaction may come from releasing cytolytic enzymes from lysosomes of liquefactive necrosis with LECs [61]. Recently, Andjelic et al. [7] through observation proposed an assumption that the fluid can enter via the holes like channels in the anterior LECs and then passing to the lens posterior pole to be a complementary role.

Together, these studies extensively support the notion that the PSC formation might be correlated with the particular inflammatory status of RP associated with the diffusion of toxins derived from the degenerated retina into the lens, which is able to interfere with the homeostatic functions of LECs leading to lens metabolic disorders and the migration of fiber cells. However, the exact interaction of these substances is unknown. Therefore, further studies about the pathogenesis of PSC development in RP are needed for providing more confirmed understanding into prevention and target treatment in RP to minimize the sight-threatening complications of the disease.

### 2.2. Other Types of Cataract

The anterior cortex of APCs in RCS rats showed numerous vesicles locating in anterior subcapsular zoom, which was considered to be caused by degeneration of elongating fibers in the bow region and subsequent damage in the superficial anterior cortex [21, 22]. According to the prevalence of APCs (21%) resembling mature cataracts (25%), Al-Ghoul and Kuszak inferred that APC formation might be a predictor of mature cataract development rather than effecting recovery by internalization of PSCs [22].

RP patients with NC formed later than PSC with the average onset age 69.6 ± 12.4 were reported. The incidence rate of NC is highest in the simplex RP (inheritance not known), about 14.8%, and 5.9% and 4.8% in ADRP and ARRP, respectively [20].

Studies on the frequency and pathological mechanism of APC and NC are scarce, showing that it is difficult to identify whether the cataract development is induced by RP or just a coexistence of disease.

## 3. Cataract Extraction

When the lens and retina are in the pathological state at the same time, it is hard to predict whether the visual acuity of RP patients after cataract surgery can increase, not to mention the higher risk of intraoperative and postoperative complications. However, favorable clinical courses and visual outcomes have been reported among all types of complicated cataract with RP patients [6, 8–14, 16, 18, 19].

### 3.1. Preoperative Examination

Accurate preoperative definition of RP could predict the cataract surgery outcome, as zonular weakness [12], shallow anterior chamber [66], or other abnormality preoperatively in RP patients will increase the rate of intraoperative and postoperative complications. Based on the following studies [8, 9, 12, 19], indications of functioning cataract surgery are suggested by a relatively healthy macula lutea in RP patients.

#### 3.1.1. Possibility of the Preoperative Zonular Weakness and Shallow Anterior Chamber

The zonular weakness and dehiscence might occur preoperatively in RP patients, perhaps, because of some toxic substances from long-term inflammatory status [12, 67, 68]. Early vitreal liquefaction and irregular vitreal scaffolding in RP patients are possibly associated [12]. Considering lots of challenges brought by zonulopathy when undergoing cataract surgery, including capsular rupture, vitreous prolapse [69], nucleus drop [70], and early anterior contraction with a resultant decrease in vision and IOL dislocation [67, 68, 71], the surgeons should be prepared to preoperatively identify these signs suggesting a possibility of zonular weakness, such as lens subluxation, zonular dialysis, or phacodonesis, which can allow for some protective measures discussed in part of capsular tension ring (CTR) [16, 70]. However, these signs usually indicate severe zonular weakness. Intraoperative assessment is considered as best to determine minor and moderate zonular weakness through surgical maneuvers and observations [70].

Several studies [72–74] described that RP might be correlated to angle closure glaucoma (ACG), and a recent study showed a genetic association between primary ACG and RP [75], although the prevalence is rare (1.03%–2.3% in RP patients [74]). In contrast, the anterior chamber angle region was not affected in an animal model of RP [76]. Like a retrospective study, Xu et al. [77] found that it may be a coincidence relationship between the patients with ACG in RP and those with ACG based on the measured similar biometric parameter among these two groups, although the anterior chamber depth was shallow in the RP group as well.

As cataract removal in patients with shallow anterior chamber is at risk of postoperative ACG leading to a detrimental effect on the visual impairment through increased IOP, combined with the attack responded to mediators and laser iridotomy, proper clinical workup and timely intervention before surgery are needed [74, 78].

#### 3.1.2. Factors Affecting Postoperative Vision

Confirmation of the important parameter associated with postoperative visual acuity and personalized preoperative risk assessment can screen the patients who are the most probably benefit from the cataract surgery. Several studies showed that the visual acuity might be correlated with the status of macular microstructures on optical coherence tomography (OCT) in RP patients [79–81]. Therefore, many studies [8, 9, 19] explored the relationship between preoperative retinal layer status and visual acuity after cataract surgery in RP patients.

The preoperative integrity of the ellipsoid zone (EZ, also known as the IS/OS line) has shown significantly better postoperative visual acuity [8, 19], perhaps, because EZ was considered as the earliest histopathological change in the outer segments of photoreceptors [79] and normal EZ means more functional macular existence suggesting that preoperative poor visual acuity is more likely the result of cataract. Similarly, the status of EZ was individually correlated with postoperative best-corrected visual acuity (BCVA) in the study of Mao et al. [9], whereas it was not significantly correlated at multiple linear regression analysis. They attributed the disparity to the different deficit order of retinal layers when measured in different timing. These studies [8, 9, 12, 19] equally reported that the preoperative central foveal thickness and external limiting membrane with OCT also showing the retinal layer status, preoperative mean deviation value on the Humphrey field analyzer (HFA 10.2) assessing the sensitivity distribution in the macular area, and preoperative BCVA have significantly correlated with final BCVA to become potential predictors of postoperative visual outcomes.

Interestingly, the mode of RP inheritance was proved to be associated with visual prognosis [10]. Auffarth et al. [82] introduced a standardized evaluation system for complicated cataract in RP, but the system's function is unknown due to lack further studies.

### 3.2. Visual Acuity Outcome of Phacoemulsification Surgery

As shown in Table 2, the disparity of mean change in the final BCVA (ranging from 0.09 to 0.47) and visual improvement (ranging from 44.8% to 96.7% eyes) [8–12, 16, 19] following phacoemulsification with IOL implantation in different studies is likely vested on different follow-up times, the degree of disease severity, sample size, potential measurement bias, and so on. Chan et al. [11] using survival analysis calculated that the mean duration of visual improvement following cataract surgery was 8.10 ± 0.83 years (95% confidence interval, 6.47 to 9.72 years), which provides proof that patients with RP could achieve a long duration of visual improvement, despite the drop of visual improvement overtimes due to the degenerative nature of the disease. However, the patient should be informed that the visual field will not improve [14] and have the possibility of unchanged (3.3%–53.6%) or worsened (0%–2.5%) visual acuity with cataract surgery [8–10, 12, 16, 19].

The study of De Rojas et al. [10] using spectral-domain OCT (SDOCT) to measure the EZ width (a marker of RP severity) demonstrated that the rate of disease progression was not associated with cataract surgery, sex, presence of CME at baseline, presence of ERM at baseline, and presence of PSC but affected by the type of inheritance of RP. In addition, OCT image repeatability significantly improved after cataract phacoemulsification in all types of cataracts reported by Garcia-Martin et al. [83].

These findings support the conclusion that if those people whose vision problems are mainly caused by visually significant cataracts rather than retinal pathology can be determined, early phacoemulsification with IOL implantation is most likely safe and effective means of visual improvement in patients with advanced RP. Further studies with relative long-term follow-up and more samples after cataract surgery are needed to determine the target patient and optimal surgery time.

### 3.3. Femtosecond Laser-Assisted Cataract Surgery

The femtosecond laser has been used in several cataract surgery stages, especially creating safe, precise, and reproducible circular capsular openings [84, 85]. The meta-analysis performed by Popovic et al. [84] for evaluating the efficacy and safety between femtosecond laser-assisted cataract surgery (FLACS) and manual cataract surgery (MCS) in 14,567 eyes indicated that patient-important visual and refractive outcomes and overall complications were not statistically significantly different, but the results regarding secondary surgical endpoints were mixed. Considering less capsular bag shrinkage resultant in a good lens position and IOL power calculations, FLACS was recognized as a surgical approach of obvious priorities in patients with complex cataracts, including cataracts with zonulopathy [86, 87]. In addition, upregulation of LEC death at the edge and inhibition of epithelial-to-mesenchymal transition with a femtosecond capsulotomy, in turn, reduced LEC proliferation, which may result in less PCO compared with traditional continuous curvilinear capsulorhexis (CCC) [85, 88, 89]. These studies supported that RP patients who usually complicate complex cataracts (with zonular weakness or other abnormality) potentially benefit from the increased safety of less zonular stress and capsular contraction with a femtosecond capsulotomy.

Although the increment of prostaglandin was detected in the aqueous humor after FLACS, the postoperative intraocular inflammation tested by laser flare photometry was actually higher in the group operated without the laser [84, 85]. In addition, FLACS has not been implicated as an essential risk factor for cystoid macular edema [85, 90]. However, complex surgical processes and expensive costs should also be well settled within the technique evolution because FLACS was not currently cost-effective than MCS [85], and the safety and effectiveness of FLACS in RP patients should be confirmed by more clinical studies.

### 3.4. Capsular Tension Ring

Given the possibility of zonular weakness, apart from careful surgical manipulations in cataract surgery to keep the integrity of zonules, including slow-motion phacoemulsification, gentle hydrodissection, viscodissection, chopping techniques, bimanual rotation of nucleus [12], meticulous cortex cleaning, and aspiration of the cortex directed tangentially [91], avoiding overinflate of the anterior chamber and a well-centered capsulorhexis [92], CTR was reported to be a safe and effective intraoperative support tool to stabilize and reinforce the zonular and postoperative IOL fixation and facilitate cataract surgery and IOL implantation to yield satisfied outcomes [71, 93–95].

Celik et al. [93] indicated that RP with zonular weakness is one of the indications for CTR implantation. In the study of Bayyoud et al. [13], they demonstrated RP eyes under phacoemulsification and IOL implantation with CTRs showed less long-term postoperative complications, such as PCO (23/52, 44%), capsular phimosis (2/52, 4%), and improved visual acuity at a mean follow-up time of 26 months. Conversely, some reported cases still exhibit late-onset subluxation or dislocation of IOL or CTR-IOL-capsular bag complex, perhaps indicating the insufficient strength of standard CTR to support a profound zonular weakness [67, 96].

Over the years, modified capsular tension ring (m-CTR, a CTR with suturing eyelets) or capsular tension segments (CTS) were introduced to provide firmer capsular bag and IOL position than standard CTR because it permits scleral-suture fixation but does not violate the integrity of the capsular bag [71, 94, 97]. In contrast, the polypropylene sutures susceptible to breakdown over time may pose some risk. A case reported that late IOL opacification occurred after using scleral suturing to deal with IOL subluxation in RP patients, indicating a potential inflammatory response to scleral suturing [98].

Few studies recently indicated that CTS or m-CTR/flanged haptic complex, a new technique with the absence of suture, flaps, and glue, may potentially overcome this disadvantage and shorten surgical time with fewer steps [69]. Besides, a successful CTR implantation needs to maintain anterior chamber depth and avoid capsular bag collapse after lens extraction conducing to maintain mydriasis but increasing stress on the zonules, so the risks and benefits of the use of CTR need to be considered [93].

A recent study introduced a method to classify the zonular weakness degree based on shifted distance of the lens during CCC in patients undergoing cataract surgery redounding to choose the optimal technique that can stabilize zonules [70]. Hence, further studies to accurately define the degree of zonular insufficiency and evaluate the exact achievable outcomes of those techniques among patients with zonular weakness in RP are required.

### 3.5. Intraocular Lens Selection

The selection of IOLs is also crucial in the eyes with RP. Optic material and optic design including haptic design and optic edge are supposed to be important factors to influence the clinical outcomes of cataract surgery.

It tends to avoid silicone IOLs owning to the nature of the IOL-LEC interaction leading to increase capsule contracture rate and the risk of IOL decentration [6, 91]. The comparison between hydrophilic and hydrophobic IOLs in the Meta-analysis by Zhao et al. [99] showed that the optic material of hydrophobic acrylic produced a lower PCO score and less neodymium-doped yttrium aluminum garnet (Nd:YAG) laser capsulotomy. In addition, compared with polymethyl methacrylate (PMMA) and silicone IOLs, hydrophobic acrylic IOLs have indicated more capsular biocompatible in vitro studies [100].

The optic edge seems to play a major role in developing anterior capsular opacification (ACO) and PCO, two types of lens capsule opacification according to the location [101]. In contrast to round-edged optic, modified square-edged led to less PCO and Nd:YAG laser capsulotomy rates [102] but slightly more ACO perhaps due to impact the LECs' migration path from anterior capsular by cell contact inhibition of sharp edge [103]. Furthermore, comparing square-edged IOL with continuous 360 degree and those with interruption at the optic-haptic junction, the PCO scores had no significant differences, but less ACO and contraction as well as glistening were seen in the continuous group after 5 years follow-up [104]. The treatment of IOL surface may play a role as well. For example, IOL binding components of extracellular matrix proteins to the IOL surface can lower PCO by promoting capsular-IOL adhesion to set up a barrier for LEC migration [105], and heparin coating IOL theoretically reduces inflammatory cell adhesion by changing the surface property to hydrophilic. Nevertheless, some studies showed contradictory results [106, 107].

There is a controversial opinion in comparing different curvature of the IOL surface [104, 108, 109] and optic design. Early studies have shown that the degree of IOL decentration and tilt in the eyes with 1-piece IOL were not significantly different compared with 3-piece IOL [110, 111], while the incidence of IOL capture was significantly higher after implanting a 1-piece IOL [112]. Some studies indicated no significant change in the amount [113], intensity, and area of PCO, the accumulative incidence of YAG treatment [114], and the percentage of anterior capsule contraction from a 3-piece to a 1-piece haptic design [110]. However, another study reported a lower level of ACO in 1-piece haptic design [115]. Previous studies reported that more forward movement in 3-piece IOL than those in 1-piece could lead to a myopic shift [116, 117]. Conversely, 3-piece IOL yields better refractive outcomes and the disparity can be explained by the rigid haptics enough to resist the capsular bag [118]. Therefore, further research is required to fully understand the functions of diverse haptic design of intraocular lens in RP patients.

One study revealed that the use of blue-light filtering IOLs could partially reduce retinal phototoxicity by blocking short-wavelength visible light [119], whereas a recent systematic review showed that it is vague whether blue-light filtering IOLs preserve macular health [120].

An excellent clinical outcome may be associated with the type of lens selected combined with the quality of surgery, but the latter ensures secure in-the-bag IOL fixation probably is more critical [121].

## 4. Complications

The higher risk of surgical complication concerning visual acuity in RP patients indicates that there may be additional pathological mechanisms compared with ARC, such as the greater level of inflammation. Complications associated with cataract surgery in RP patients can occur from phototoxic retinal damage during the surgery, postoperative PCO, CCS, PCME, increased IOP, and IOL subluxation or dislocation [6, 8, 10, 12, 13, 16, 17]. It has been associated with inflammation response of progressive retinal degeneration and foreign matter, surgery trauma, zonular weakness, and degenerative aging process [12, 34, 68, 105, 122–124].

### 4.1. Phototoxic Retinal Damage

Some clinical evidence of reported cases and experimental evidence of animal models supported that the microscope light has damaging effects on the retina of RP patients including unexpected visual results, although definitive proof is lacking [125–128]. It is most prevalent in ADRP patients due to 30% carrying a mutation in the RHO gene susceptible to light damage [128].

There are numerous risks of light damage in cataract surgery including immobilized eyes, longer exposure time, an intense level [129], and wavelengths between 400 nm and 500 nm of light [130]. Although minimizing light exposures and density during ophthalmic surgical procedures may be necessary, making a clinical judgment by practitioners between the risk of brighter light and the consequence of insufficient light is more important [129]. Using intracameral illumination rather than the microscope illumination during cataract surgery can reduce light exposure reaching a patient's retina [131].

One study to evaluate the near-infrared (NIR) operating microscopy (NIOM) system using NIR wavelength (850 to 1300 nm) as the illumination source instead of visible light that produces the majority of light damage showed that it seems useful for obtaining good visual acuity. However, NIR wavelength safety needs more definite evidence when used in clinical surgery [130].

### 4.2. Posterior Capsule Opacification

PCO (67%–95% frequency) is the most frequently common sight-threaten complication following cataract surgery, which is abnormally stronger in RP eyes than in healthy eyes (20%–40% [105]) and about 17%–52% of RP patients required further Nd:YAG laser treatment—the primary therapy to deal with the secondary opaque visual axis [6, 8, 10, 12, 13, 16, 122]. The cumulative PCO rate in RP after the third postoperative year was up to 70.7%, and about 25% of RP patients required further Nd:YAG laser treatment showed in the study of Auffarth et al. [132]. The PCO is recognized as the result of capsular fibrosis associated with migration, proliferation, and epithelial-mesenchymal transition of LECs [122], which might be aggravated by the higher cytokines in RP eyes [123]. The PCO development is age-dependent, with a higher rate in young patients [133], probably since the wound healing response differs from the older patients [134]. As mentioned before, most cataract surgery in RP patients performed at a young age partially explained the high risk for PCO.

Various additional techniques and IOL design and material (mentioned in part of IOL selection) can lower PCO incidence through interfering with the biological processes in LECs [105]. The adjunct technique, such as capsule polishing, simple aspiration, ultrasound aspiration, and osmolysis, is recommended to apply to eyes with RP because LEC removal can reduce or delay fibrosis and shrinkage of the anterior capsule. However, clearing LECs completely is tough, and additional surgical time and potential activation of LEC proliferation may also occur [135].

CTRs may be applied against the fibrosis and shrinkage of the anterior capsule in favor of LEC removal [12], and the sharp bend of CTRs helps to suppress LEC proliferation [135]. Opening of the anterior capsule by CCC might result in less amount of PCO than those with rim tears, linear or can-opener capsulotomy, and envelope capsulectomy [136, 137], whereas a study showed that the PCO score was slightly higher in CCC than femtosecond laser-assisted anterior capsulotomy [89]. Considering these methods conducive to slow down its progress rather than wholly stop, fundamental disruption of LEC proliferation and metaplasia on etiology needs further study.

### 4.3. Capsule Contraction Syndrome

**Figure 3:**
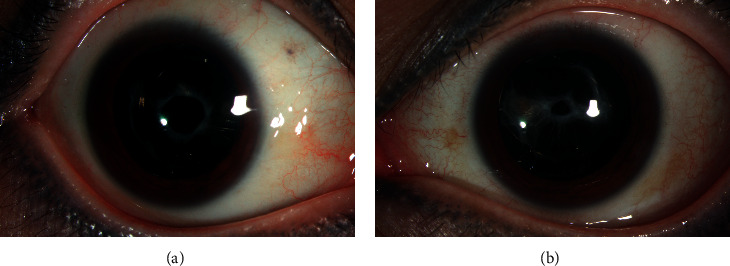
Example of a patient occurred bilateral anterior capsular opacification (ACO) or anterior capsular contraction following phacoemulsification with hydrophilic IOLs. (a) ACO occurred in the right eyes after 2.5 months. (b) ACO occurred in the left eyes after 3 months.

CCS is defined as an exaggerated reduction in anterior capsulectomy and capsular bag diameter after extracapsular cataract surgery [124] (Figure 3). The fibrotic reaction of residual LECs associated with ACO [101] and extracellular matrix (ECM) disorder might together work on CCS's pathogenesis in RP [34, 68, 123, 124]. Recently, a new potential target of stimulating LEC proliferation, tenascin‐C (TNC), was found in a proteomic analysis of the aqueous humor between senile cataract and RP patients with cataracts to be a candidate protein underlying capsular shrinkage pathogenesis [138].

The frequency of the postoperative CCS cases (about 10%–23% [6, 8]) is extensive. In addition, the long-term consequences reported by the following studies are also quite depressive. Anterior capsule opening area decreased to pupillary may cause visual acuity deterioration and blur vision [139]. The traction on the ciliary processes seems to result in hypotony [67]. CCS may also continuously exacerbate into more severe IOL dislocation leading to refractive changes and glare [17, 139], followed by retinal and ciliary body detachment if without intervention [124].

The postoperative tightly clinical observation is necessary for patients to timely discover the occurrence of fibrosis and extensive contraction of anterior capsule and treat with early YAG laser anterior capsulotomy [140] to prevent shrinkage further because late intervention with high risk of IOL dislocation may not help either nearly as much [124]. Wilde et al. [140] recommended that YAG anterior capsulotomy can be first performed in a spoke-like pattern, radiating perpendicularly from the edge of the capsule margin, which is a safe and effective technique for reducing the formation of free-floating remnants caused by circular anterior capsulotomy, followed by further radial YAG laser capsulotomy or surgical approaches if the method fails. Moreover, caution should be taken into Nd:YAG capsulotomy in RP patients to avoid further zonular loosening [91].

Based on the interplay between zonular weakness and CCS [67, 68, 71, 96], the patients are deemed to benefit from larger capsulorhexis and small radial incisions intraoperatively [17, 92]. The additional technique to industriously remove residual LECs mentioned in the part of PCO also seems useful to decrease lens fibrosis.

### 4.4. Pseudophakic Cystoid Macular Edema

PCME (also called Irvine–Gass syndrome) consists of the maldistribution of retinal intravascular fluid within the macular following cataract surgery leading to suboptimal visual acuity, which was first recognized by Irvine in 1953 [141, 142]. PCME can be found in RP (13.3%–32% [6, 10, 16]), which was more extensive than those without (1.17–4.2% [90]) on account of an inflammatory process to increase vascular permeability and disrupt blood-ocular barriers [46–48, 142]. Flach's study showed that some PCME patients were unresponsive to anti-inflammatory drugs but responsive to acetazolamide treatment that can improve the pumping function of the pigmented retinal epithelium (RPE) [142]. RP patients also interfere with the function of RPE [1], which perhaps exacerbates PCME formation to become a potential mechanism.

Against the pathogenesis of PCME, prophylactic anti-inflammatory interventions significantly reduce the risk of developing CME after cataract surgery [90]. The administration regimens mainly include the sole use of topical nonsteroidal anti-inflammatory drugs (NSAIDs), topical corticosteroids, or both at the same time, but the optimal regimens are hard to conclude [143] and mainly depend on individualization. In a systematic review, topical NSAIDs are more effective than topical steroids in preventing PCME after uncomplicated phacoemulsification with high-quality evidence [144]. When PCME has occurred, patients treated with a combination of topical corticosteroids and NSAIDs will resolve it in a shorter period [90]. What is more, the side effects of drug should be taken into consideration, especially corticosteroids which presumably induce IOP increment [145].

The possibility of photoreceptor loss caused by longer duration of PCME indicated the necessity to prompt diagnosis and adequate treatment, although the majority of acute PCME resolves spontaneously [90]. Davies and Pineda recommended that all patients were instructed to use a topical NSAID and carbonic anhydrase inhibitor for 3 months after cataract surgery or YAG capsulotomy [16] by some studies cited in the literature review [90] indicating that the risk of PCME could be further reduced if treatment is continued for 3 months postoperatively.

### 4.5. Other Complications

RP was reported to be one of the predisposing factors of late spontaneous IOL-capsular bag complex dislocation [91, 146], which is defined as occurring 3 months or later following cataract surgery, mainly on account of zonular weakness and capsular contraction [67, 68, 91, 96, 139, 140, 147–149].

One study reported that the postoperative increased IOP occurred in 10% of cases, so surgeons should be aware of the possibility and the need for regular examination to timely treat with typical antiglaucoma mediators [13]. A randomized clinical study showed that the patients with glaucoma prophylactically administered oral acetazolamide 1 hour before surgery can significantly reduce the IOP elevation from 1 to 24 hours, indicating that the drug acts rapidly and effectively, while it is unclear whether the eyes with RP have equal effect on this count [150].

## 5. Conclusion

RP is a leading cause of visual disability and is often complicated by cataracts. Patients' visual acuity and life quality are affected in RP patients with complicated cataract, whose cataract surgery with the higher risk factors faces many challenges. Considering the visual gain realized for most RP patients when taking closely both preoperative and postoperatively monitor and the use of specialized adjunctive devices intraoperatively, phacoemulsification with IOL implantation seems to be the preferred method of extraction in visual evidential cataracts, even in the advanced RP. FLACS to treat cataracts in RP also has great potential in the future. In addition, the long-term sequela of ocular inflammation plays a role in disease progression and many surgery complications with RP, including PCO, CCS, and PCME. Therefore, it is wise to use prophylactic anti-inflammatory drugs to prevent these adverse affairs and the best regime needs further exploration. Complicated cataract in RP usually needs a surgical solution, which is hard to evitable due to the high incidence, so further works are required to determine the conclusive evidence of the pathogenesis to identify targeted and effective therapeutics instead of surgery, and specific prevention to limit the occurrence of surgical complication.

## 6. Method of Literature Search

This review includes thorough publication on retinitis pigmentosa with complicate cataract published from 1953 through 2020. PubMed and MEDLINE databases were searched using the following terms in various combinations: complicated cataract with retinitis pigmentosa, posterior subcapsular cataract, anterior polar cataract, cataract surgery, postoperative complications, and management or treatment. Surgical procedures as discussed in the review were also used as search terms. Articles related to the morphology, pathogenesis, treatment, complications, and prevention of retinitis pigmentosa with complicated cataract were included. References were also obtained from citations in papers found in the original search. Relevant non-English language articles were obtained when translation was available.

## Figures and Tables

**Table 1:** Changes of cytokines between RP patients and the control group in different fluid.

Author	Year	Test group	Control group	IL	Chemokines	GF	MMP and other cytokines
Okita et al. [35]	2020	Serum in RP patients	Serum in without RP	IL-2↑, IL-10↑, IL-17↑	IL-8↑, RANTES↑	NA	n.s.
Lu et al. [34]	2020	AH in RP patients	AH in ARC	n.s.	IL-8↑, MCP-1↑, IP-10↑	HGF↑, PDGF-AA↑	MMP-2↑, MMP3↑, MMP-7↑, MMP-8↑, PAI-1↑, TSP-2↑, BMP-4↓
Ten Berge et al. [33]	2019	IOF in RP patients	RP paired serum	IL-2↑, IL-6ra↓, IL-6↑, IL-23↓	MCP-1↑, TARC↓	PlGF↑, VEGF↓	n.s.
Yoshida et al. [31]	2013	AH in RP patients	AH in cataract	n.s.	IL-8↑, TARC↑, MCP-1↑	NA	n.s.
		Vitreous fluid in RP patients	Vitreous fluid in iERM patients	IL-1*α*↑, IL-1*β*↑, IL-2↑, IL-4↑, IL-6↑, IL-10↑	IL-8↑, TARC↑, MCP-1↑, MCP-2↑, GRO-*α*↑, I-309↑, IP-10↑	NA	IFN-*γ*↑
Salom et al. [37]	2010	AH in RP patients	AH in cataract	NA	NA	HGF↑	NA
Salom et al. [36]	2008	AH in RP patients	AH in cataract	NA	NA	VEGF-A↓	NA

All data in the list were considered statistically significant (*p*-value < 0.05). RP = retinitis pigmentosa; IL = interleukin; GF = growth factor; MMP = matrix metalloproteinase; RANTES = regulated activation normal T-cell expressed and secreted; NA = not available; n.s. = not statistically significant (*p* > 0.05); AH = aqueous humor; ARC = age-related cataract; MCP-1 = monocyte chemotactic protein 1; IP-10 = interferon *γ* inducible protein 10; HGF = hepatocyte growth factor; PDGF-AA = platelet-derived growth factor AA; PAI-1 = plasminogen activator inhibitor-1; TSP-2 = thrombospondin-2; BMP-4 = bone morphogenetic protein-4; IOF = intraocular fluid; TARC = thymus- and activation-regulated chemokine; PlGF = placental growth factor; VEGF = vascular endothelial growth factor; iERM = idiopathic epiretinal membrane; Gro-*α* = growth related oncogene-*α*; IFN-*γ* = interferon gamma.

**Table 2:** Overview of phacoemulsification with IOL implantation outcomes in RP patients for the last decade.

Author	Mao et al. [9]	De Rojas et al. [10]	Davies and Pineda [16]	Chan et al. [11]	Yoshida et al. [8]	Nakamura et al. [19]	Dikopf et al. [12]
Year	2018	2017	2017	2017	2015	2015	2013
Number	109 eyes	19 eyes	30 eyes	67 eyes	56 eyes	58 eyes	80 eyes
Surgery age (years) mean ± SD	53.4 ± 10.3	51 ± 13	52.4 ± 13.7	59.2 ± 12.3	62.6 ± 10.4	29–83 (range)	48.9 (mean)
Mean follow-up time	3 months	259 days (median)	3.7 ± 3.3 months	6.9 ± 4.4 years	37.5 ± 22.6 months	3 months	23.3 months
Baseline BCVA (log MAR) mean ± SD	0.8 ± 0.59	0.33 ± 0.20	1.09 ± 0.69	1.27 ± 0.42	0.76 ± 0.65	0.81 ± 0.51	1.23 ± 0.99
Postoperative BCVA (log MAR) mean ± SD	0.45 ± 0.41 (final)	0.19 ± 0.17 (final)	0.61 ± 0.45 (1 month)	0.92 ± 0.49 (3 months) 0.97 ± 0.53 (1 year) 1.18 ± 0.49 (final)	0.42 ± 0.55 (6 months) 0.45 ± 0.53 (final)	0.34 ± 0.43 (final)	0.81 ± 0.87 (3 months)
Change in final BCVA (log MAR)	0.35	0.14	NA	0.09	0.31	0.47	NA
Improved VA	52/109, 47.7%	17/19, 89.0%	29/3., 96.7%	30/67, 44.8%	26/56, 46.4%	37/58, 63.8%	70/80, 87.5%
Unchanged VA	57/109, 52.3%	2/19, 11.0%	1/30, 3.3%	37/67, 55.2%	30/56, 53.6%	21/58, 36.2%	8/80, 10.0%
Worsened VA	0%	0%	0%		0%	0%	2/80, 2.5%
Complication	NA	PCME (6/19, 32%) PCO (18/19, 95%)	PCME (4/30, 13.3%) PCO (20/30, 66.7%) nd: YAG laser capsulotomy (5/30, 16.7%)	NA	PCO (47/56, 83.9%) Nd: YAG laser capsulotomy (23/56, 41.1%) CCS (13/56, 23.2%)	None	PCO (66/80, 82.5%) YAG laser capsulotomy (42/80, 52.5%) IOL dislocation (2/80, 2.5%)

RP = retinitis pigmentosa; BCVA = best-corrected visual acuity; log MAR = logarithm of the minimum angle of resolution; SD = standard deviation; VA = visual acuity; PCME = pseudophakic cystoid macular edema; PCO = posterior capsular opacification; Nd:YAG = neodymium-doped yttrium aluminium garnet; IOP = intraocular pressure; IOL = intraocular lens; CCS = capsular contraction syndrome; NA = not available.

## Data Availability

No data were used to support this study.
